# Supplementary material for: Prevalence of unmasked and improperly masked behavior in indoor public areas during the COVID-19 pandemic: Analysis of a stratified random sample from Louisville, Kentucky
Source: PLoS One. 2021 Jul 28;16(7):e0248324. doi: 10.1371/journal.pone.0248324 (PMC8318281; doi:10.1371/journal.pone.0248324)
Supplement: S1 File — (DOCX) [file pone.0248324.s001.docx]

**S1 File.**

**S1 Table.** Constructed city districts by zip codes

| **Districts** | **Zip Codes** |
| --- | --- |
| South & South West | 40272, 40258, 40118, 40229 |
| West Center | 40208, 40215, 40216 |
| North West | 40212, 40211, 40210, 40202, 40203 |
| North Center | 40206, 40204, 40217, 40205, 40207 |
| Central | 40209, 40213, 40214, 40219, 40218 |
| South East | 40220, 40299, 40291, 40228 |
| East & North East | 40023, 40245, 40243, 40223, 40241, 40242, 40222, 40059 |

**S2 Table.** Louisville population characteristics by zip code and district

|  |  |  | **Census** |  |  |  |  | **Percentage** | **Percentage** |  | **Median** |
| --- | --- | --- | --- | --- | --- | --- | --- | --- | --- | --- | --- |
|  |  | **Zip** | **2010** | **Age Group (%)** | | | | **White** | **Black** | **Percentage** | **Household** |
|  | **District** | **Code** | **Population** | **0-18** | **19-44** | **45-64** | **65+** | **Non-Hispanic** | **Non-Hispanic** | **Hispanic** | **Income (2018)** |
| 1 | South & South West | 40118 | 9,724 | 26% | 35% | 27% | 11% | 86% | 2% | 9% | 52,848 |
|  |  | 40229 | 36,852 | 28% | 38% | 24% | 10% | 87% | 6% | 4% | 58,685 |
|  |  | 40272 | 37,394 | 26% | 33% | 27% | 14% | 91% | 4% | 3% | 55,052 |
|  |  | 40258 | 26,465 | 26% | 33% | 27% | 14% | 84% | 11% | 2% | 51,184 |
|  |  | *Weighted Average* | | *27%* | *35%* | *26%* | *12%* | *88%* | *6%* | *4%* | *55,143* |
| 2 | West Center | 40208 | 13,227 | 20% | 53% | 21% | 6% | 61% | 29% | 4% | 28,026 |
|  |  | 40215 | 22,287 | 29% | 36% | 26% | 9% | 57% | 32% | 7% | 32,306 |
|  |  | 40216 | 40,746 | 25% | 32% | 29% | 14% | 60% | 34% | 3% | 43,358 |
|  |  | Weighted Average | | 25% | 37% | 27% | 11% | 59% | 33% | 4% | 37,469 |
| 3 | North West | 40202 | 6,772 | 11% | 54% | 28% | 6% | 38% | 54% | 3% | 17,940 |
|  |  | 40203 | 19,694 | 28% | 36% | 24% | 12% | 31% | 64% | 2% | 16,862 |
|  |  | 40210 | 14,822 | 30% | 31% | 26% | 12% | 7% | 89% | 1% | 23,330 |
|  |  | 40211 | 22,612 | 31% | 30% | 26% | 13% | 4% | 93% | 1% | 27,565 |
|  |  | 40212 | 17,685 | 29% | 31% | 28% | 11% | 36% | 60% | 1% | 24,957 |
|  |  | *Weighted Average* | | *28%* | *34%* | *26%* | *12%* | *21%* | *75%* | *1%* | *22,848* |
| 4 | North Center | 40204 | 14,236 | 15% | 47% | 27% | 11% | 85% | 9% | 2% | 52,179 |
|  |  | 40205 | 23,678 | 19% | 35% | 31% | 16% | 93% | 3% | 2% | 76,802 |
|  |  | 40206 | 18,865 | 17% | 42% | 28% | 13% | 84% | 9% | 3% | 55,156 |
|  |  | 40207 | 29,745 | 21% | 32% | 28% | 18% | 90% | 3% | 3% | 78,750 |
|  |  | 40217 | 12,507 | 17% | 44% | 27% | 12% | 86% | 7% | 2% | 50,498 |
|  |  | *Weighted Average* | | *18%* | *38%* | *28%* | *15%* | *88%* | *6%* | *2%* | *66,402* |
| 5 | Central | 40213 | 16,796 | 23% | 37% | 27% | 14% | 71% | 18% | 8% | 42,437 |
|  |  | 40214 | 45,291 | 25% | 37% | 26% | 12% | 68% | 14% | 9% | 41,449 |
|  |  | 40218 | 31,658 | 26% | 37% | 25% | 12% | 47% | 39% | 8% | 44,587 |
|  |  | 40219 | 38,032 | 25% | 36% | 25% | 14% | 63% | 21% | 12% | 46,931 |
|  |  | *Weighted Average* | | *25%* | *37%* | *26%* | *13%* | *62%* | *23%* | *9%* | *43,911* |
| 6 | South East | 40220 | 33,109 | 21% | 34% | 28% | 17% | 76% | 14% | 5% | 62,366 |
|  |  | 40228 | 15,743 | 26% | 34% | 27% | 14% | 77% | 15% | 4% | 66,687 |
|  |  | 40291 | 35,427 | 25% | 35% | 28% | 12% | 82% | 10% | 4% | 70,234 |
|  |  | 40299 | 38,371 | 26% | 33% | 29% | 13% | 85% | 8% | 4% | 76,587 |
|  |  | *Weighted Average* | | *24%* | *34%* | *28%* | *14%* | *81%* | *11%* | *4%* | *69,642* |
| 7 | East & North East | 40023 | 4,118 | 27% | 31% | 34% | 9% | 94% | 2% | 2% | 73,775 |
|  |  | 40059 | 16,708 | 30% | 24% | 34% | 12% | 87% | 4% | 2% | 142,354 |
|  |  | 40222 | 21,359 | 19% | 34% | 26% | 20% | 82% | 7% | 4% | 73,220 |
|  |  | 40223 | 22,011 | 25% | 29% | 31% | 15% | 82% | 9% | 4% | 78,076 |
|  |  | 40241 | 28,988 | 24% | 32% | 29% | 15% | 78% | 10% | 4% | 78,331 |
|  |  | 40242 | 10,930 | 23% | 34% | 27% | 16% | 82% | 8% | 5% | 68,004 |
|  |  | 40243 | 10,210 | 22% | 28% | 28% | 22% | 87% | 5% | 4% | 73,448 |
|  |  | 40245 | 30,109 | 29% | 34% | 29% | 8% | 81% | 9% | 3% | 114,092 |
|  |  | *Weighted Average* | | *25%* | *31%* | *29%* | *14%* | *82%* | *8%* | *4%* | *91,141* |

*Note*: Authors calculations using population information from U.S. Census 2010 (<https://www.census.gov/data/tables/time-series/demo/popest/2010s-counties-total.html>) and household income data from American Community Surveys (<https://www.census.gov/topics/income-poverty/income/data/tables/acs.html>).

**S3 Table.** Populations of public areas in each surveying cluster, characterized by size, city district, and retail trade group

|  |  | Retail Trade Groups | | | |
| --- | --- | --- | --- | --- | --- |
| City District | Total | 1 | 2 | 3 | 4 |
| Square Footage Less than 1,499 | | | | | |
| South & South West | 107 | 20 | 10 | 9 | 68 |
| West Center | 112 | 9 | 18 | 22 | 63 |
| North West | 163 | 12 | 23 | 40 | 88 |
| North Center | 234 | 33 | 31 | 19 | 151 |
| Central | 207 | 37 | 25 | 38 | 107 |
| South East | 168 | 31 | 15 | 23 | 99 |
| East & North East | 195 | 27 | 43 | 20 | 105 |
| Square Footage between 1,500 and 2,499 | | | | | |
| South & South West | 92 | 12 | 16 | 28 | 36 |
| West Center | 123 | 17 | 26 | 42 | 38 |
| North West | 190 | 25 | 42 | 59 | 64 |
| North Center | 261 | 38 | 44 | 63 | 116 |
| Central | 237 | 42 | 44 | 69 | 82 |
| South East | 220 | 39 | 46 | 46 | 89 |
| East & North East | 237 | 34 | 45 | 53 | 105 |
| Square Footage between 2,500 and 4,999 | | | | | |
| South & South West | 70 | 19 | 16 | 19 | 16 |
| West Center | 56 | 13 | 11 | 16 | 16 |
| North West | 111 | 17 | 25 | 24 | 45 |
| North Center | 181 | 43 | 48 | 28 | 62 |
| Central | 160 | 34 | 44 | 37 | 45 |
| South East | 160 | 39 | 36 | 35 | 50 |
| East & North East | 216 | 48 | 55 | 29 | 84 |
| Square Footage Greater than or Equal to 5,000 | | | | | |
| South & South West | 91 | 18 | 36 | 11 | 26 |
| West Center | 82 | 10 | 28 | 22 | 22 |
| North West | 87 | 20 | 25 | 9 | 33 |
| North Center | 236 | 40 | 71 | 29 | 96 |
| Central | 221 | 55 | 64 | 31 | 71 |
| South East | 145 | 38 | 33 | 19 | 55 |
| East & North East | 286 | 57 | 76 | 38 | 115 |

*Retail Trade Group 1*: SIC codes 52xxxx (Building Materials, Hardware, Garden Supply, and Mobile Home Dealers) and 57xxxx (Home Furniture, Furnishings and Equipment Stores)

*Retail Trade Group 2*: SIC 53xxxx (General Merchandise Stores) and 56xxxx (Apparel and Accessory Stores)

*Retail Trade Group 3*: SIC codes 54xxxx (Food Stores)

*Retail Trade Group 4*: SIC codes 59xxxx (Miscellaneous Retail)

**S4 Table.** Numbers of randomly selected public areas in each surveying cluster, characterized by size, city district, and retail trade group

|  |  | Retail Trade Groups | | | |
| --- | --- | --- | --- | --- | --- |
| City District | Total | 1 | 2 | 3 | 4 |
| Square Footage Less than 1,499 | | | | | |
| South & South West | 14 | 3 | 3 | 3 | 5 |
| West Center | 14 | 3 | 3 | 3 | 5 |
| North West | 15 | 3 | 3 | 4 | 5 |
| North Center | 16 | 4 | 4 | 3 | 5 |
| Central | 16 | 4 | 3 | 4 | 5 |
| South East | 15 | 4 | 3 | 3 | 5 |
| East & North East | 16 | 4 | 4 | 3 | 5 |
| Square Footage between 1,500 and 2,499 | | | | | |
| South & South West | 14 | 3 | 3 | 4 | 4 |
| West Center | 15 | 3 | 4 | 4 | 4 |
| North West | 17 | 3 | 4 | 5 | 5 |
| North Center | 18 | 4 | 4 | 5 | 5 |
| Central | 18 | 4 | 4 | 5 | 5 |
| South East | 19 | 4 | 5 | 5 | 5 |
| East & North East | 19 | 4 | 5 | 5 | 5 |
| Square Footage between 2,500 and 4,999 | | | | | |
| South & South West | 12 | 3 | 3 | 3 | 3 |
| West Center | 12 | 3 | 3 | 3 | 3 |
| North West | 14 | 3 | 3 | 3 | 5 |
| North Center | 18 | 4 | 5 | 4 | 5 |
| Central | 17 | 4 | 4 | 4 | 5 |
| South East | 17 | 4 | 4 | 4 | 5 |
| East & North East | 19 | 5 | 5 | 4 | 5 |
| Square Footage Greater than or Equal to 5,000 | | | | | |
| South & South West | 14 | 3 | 4 | 3 | 4 |
| West Center | 13 | 3 | 4 | 3 | 3 |
| North West | 13 | 3 | 3 | 3 | 4 |
| North Center | 18 | 4 | 5 | 4 | 5 |
| Central | 19 | 5 | 5 | 4 | 5 |
| South East | 16 | 4 | 4 | 3 | 5 |
| East & North East | 19 | 5 | 5 | 4 | 5 |

*Retail Trade Group 1*: SIC codes 52xxxx (Building Materials, Hardware, Garden Supply, and Mobile Home Dealers) and 57xxxx (Home Furniture, Furnishings and Equipment Stores)

*Retail Trade Group 2*: SIC 53xxxx (General Merchandise Stores) and 56xxxx (Apparel and Accessory Stores)

*Retail Trade Group 3*: SIC codes 54xxxx (Food Stores)

*Retail Trade Group 4*: SIC codes 59xxxx (Miscellaneous Retail)

**S5 Table.** The difference between the share of selected PAs and human population shares by district

|  | Percentage | Percentage | Number of | Percentage of | Percentage of Selected PAs | |
| --- | --- | --- | --- | --- | --- | --- |
| County | of County | of PA | Selected | Selected | Minus the Percentage of | |
| District | Population | Population | PAs | PAs | Human Population | PA Population |
| South & South West | 14% | 8% | 54 | 12% | -1.9% | 4.3% |
| West Center | 10% | 8% | 54 | 12% | 2.1% | 4.1% |
| North West | 11% | 12% | 59 | 13% | 2.2% | 1.3% |
| North Center | 13% | 20% | 70 | 16% | 2.7% | -4.0% |
| Central | 17% | 18% | 70 | 16% | -1.3% | -2.1% |
| South East | 16% | 15% | 67 | 15% | -1.0% | 0.1% |
| East & North East | 19% | 20% | 73 | 16% | -2.7% | -3.8% |
| Sum | 100% | 100% | 447 | 100% | 0.0% | 0.0% |

**S6 Table**: The survey questionnaire

| **Section 1: PA Information** | | | | | | | | | | | | | | | | | |
| --- | --- | --- | --- | --- | --- | --- | --- | --- | --- | --- | --- | --- | --- | --- | --- | --- | --- |
| 1.1 | Observation Number (Transfer to the Assignment Sheet) | | | | | | | | | | | | | *Automatically Generated* | | | |
|  | | | | | | | | | | | | | | | | | |
| 1.2 | Date of Survey | | | | | | | | | | | | | *Automatically Generated* | | | |
|  | | | | | | | | | | | | | | | | | |
| 1.3 | Time of Survey | | | | | | | | | | | | | *Automatically Generated* | | | |
|  | | | | | | | | | | | | | | | | | |
| 1.4 | PA Name | | | | | | | | | | | | |  | | | |
|  | | | | | | | | | | | | | | | | | |
| 1.5 | PA Unique ID (Bring from the Assignment Sheet) | | | | | | | | | | | | |  | | | |
|  | | | | | | | | | | | | | | | | | |
| **Section 2: Observing Visitors** | | | | | | | | | | | | | | | | | |
| 2.1 | # OF OBSERVED VISITORS **[IN A ROW. NO MORE THAN 10]** | | | | | | | | | | | | | | | | |
|  |  |  | |  | |  | |  |  |  | |  | | |  | |  |
|  | | | | | | | | | | | | | | | | | |
| 2.2 | AMONG THE OBSERVED VISITORS: # UNmasked | | | | | | | | | | | | | | | | |
|  |  |  | |  | |  | |  |  |  | |  | | |  | |  |
|  | | | | | | | | | | | | | | | | | |
| 2.3 | AMONG THE OBSERVED VISITORS: # INCORRECTLY Masked | | | | | | | | | | | | | | | | |
|  |  |  | |  | |  | |  |  |  | |  | | |  | |  |
|  | | | | | | | | | | | | | | | | | |
| 2.4 | Sex and Age of UNmasked Visitors **[FIRST THREE]** | | | | | | | | | | | | | | | | |
|  |  | | Sex | | | | Age | | | | | | | | | | |
|  |  | | Male | | Female | | 0−18 | | 19−24 | | 25−44 | | 45−64 | | | 65+ | |
|  | Visitor 1 | |  | |  | |  | |  | |  | |  | | |  | |
|  | Visitor 2 | |  | |  | |  | |  | |  | |  | | |  | |
|  | Visitor 3 | |  | |  | |  | |  | |  | |  | | |  | |
|  | | | | | | | | | | | | | | | | | |
| 2.5 | Sex and Age of INCORRECTLY Masked Visitors **[FIRST THREE]** | | | | | | | | | | | | | | | | |
|  |  | | Sex | | | | Age | | | | | | | | | | |
|  |  | | Male | | Male | | 0−18 | | 19−24 | | 25−44 | | 45−64 | | | 65+ | |
|  | Visitor 1 | |  | |  | |  | |  | |  | |  | | |  | |
|  | Visitor 2 | |  | |  | |  | |  | |  | |  | | |  | |
|  | Visitor 3 | |  | |  | |  | |  | |  | |  | | |  | |
|  | | | | | | | | | | | | | | | | | |
| **Section 3: Observing Staff** | | | | | | | | | | | | | | | | | |
| 3.1 | # OF OBSERVED STAFF. **[IN A ROW. NO MORE THAN 10]** | | | | | | | | | | | | | | | | |
|  |  |  | |  | |  | |  |  |  | |  | | |  | |  |
|  | | | | | | | | | | | | | | | | | |
| 3.2 | AMONG THE OBSERVED STAFF: # UNmasked | | | | | | | | | | | | | | | | |
|  |  |  | |  | |  | |  |  |  | |  | | |  | |  |
|  | | | | | | | | | | | | | | | | | |
| 3.3 | AMONG THE OBSERVED STAFF: # INCORRECTLY Masked | | | | | | | | | | | | | | | | |
|  |  |  | |  | |  | |  |  |  | |  | | |  | |  |
|  | | | | | | | | | | | | | | | | | |
| 3.4 | Sex and Age of UNMASKED Staff. **[FIRST THREE]** | | | | | | | | | | | | | | | | |
|  |  | | Sex | | | | Age | | | | | | | | | | |
|  |  | | Male | | Female | | 0−18 | | 19−24 | | 25−44 | | 45−64 | | | 65+ | |
|  | Visitor 1 | |  | |  | |  | |  | |  | |  | | |  | |
|  | Visitor 2 | |  | |  | |  | |  | |  | |  | | |  | |
|  | Visitor 3 | |  | |  | |  | |  | |  | |  | | |  | |
|  | | | | | | | | | | | | | | | | | |
| 3.5 | Sex and Age of INCORRECTLY Masked Staff. **[FIRST THREE]** | | | | | | | | | | | | | | | | |
|  |  | | Sex | | | | Age | | | | | | | | | | |
|  |  | | Male | | Male | | 0−18 | | 19−24 | | 25−44 | | 45−64 | | | 65+ | |
|  | Visitor 1 | |  | |  | |  | |  | |  | |  | | |  | |
|  | Visitor 2 | |  | |  | |  | |  | |  | |  | | |  | |
|  | Visitor 3 | |  | |  | |  | |  | |  | |  | | |  | |

**S7 Table.** Numbers of observed public areas in each surveying cluster, characterized by size, city district, and retail trade group

|  |  | Retail Trade Groups | | | |
| --- | --- | --- | --- | --- | --- |
| City District | Total | 1 | 2 | 3 | 4 |
| Square Footage Less than 1,499 | | | | | |
| South & South West | 7 | 1 | 1 | 3 | 2 |
| West Center | 11 | 3 | 0 | 3 | 5 |
| North West | 6 | 1 | 0 | 4 | 1 |
| North Center | 16 | 2 | 5 | 5 | 4 |
| Central | 8 | 4 | 0 | 4 | 0 |
| South East | 5 | 2 | 0 | 2 | 1 |
| East & North East | 9 | 2 | 0 | 2 | 5 |
| Square Footage between 1,500 and 2,499 | | | | | |
| South & South West | 9 | 3 | 1 | 4 | 1 |
| West Center | 11 | 3 | 1 | 4 | 3 |
| North West | 10 | 1 | 0 | 5 | 4 |
| North Center | 12 | 2 | 3 | 6 | 1 |
| Central | 11 | 4 | 2 | 5 | 0 |
| South East | 13 | 1 | 1 | 10 | 1 |
| East & North East | 8 | 2 | 0 | 3 | 3 |
| Square Footage between 2,500 and 4,999 | | | | | |
| South & South West | 11 | 2 | 3 | 3 | 3 |
| West Center | 12 | 2 | 3 | 3 | 4 |
| North West | 3 | 0 | 0 | 2 | 1 |
| North Center | 17 | 1 | 5 | 9 | 2 |
| Central | 9 | 2 | 3 | 4 | 0 |
| South East | 15 | 2 | 2 | 8 | 3 |
| East & North East | 15 | 0 | 2 | 7 | 6 |
| Square Footage Greater than or Equal to 5,000 | | | | | |
| South & South West | 24 | 4 | 7 | 6 | 7 |
| West Center | 16 | 2 | 6 | 5 | 3 |
| North West | 13 | 1 | 5 | 3 | 4 |
| North Center | 26 | 4 | 11 | 6 | 5 |
| Central | 21 | 8 | 9 | 4 | 0 |
| South East | 29 | 4 | 11 | 6 | 8 |
| East & North East | 35 | 8 | 10 | 7 | 10 |

*Retail Trade Group 1*: SIC codes 52xxxx (Building Materials, Hardware, Garden Supply, and Mobile Home Dealers) and 57xxxx (Home Furniture, Furnishings and Equipment Stores)

*Retail Trade Group 2*: SIC 53xxxx (General Merchandise Stores) and 56xxxx (Apparel and Accessory Stores)

*Retail Trade Group 3*: SIC codes 54xxxx (Food Stores)

*Retail Trade Group 4*: SIC codes 59xxxx (Miscellaneous Retail)

**S8 Table.** The difference between the share of observed PAs and human population shares by district

|  | Percentage | Percentage | Number of | Percentage of | Percentage of Observed PAs | |
| --- | --- | --- | --- | --- | --- | --- |
| County | of County | of PA | Observed | Observed | Minus the Percentage of | |
| District | Population | Population | PAs | PAs | Human Population | PA Population |
| South & South West | 14% | 8% | 51 | 13% | -0.6% | 5.6% |
| West Center | 10% | 8% | 50 | 13% | 3.1% | 5.1% |
| North West | 11% | 12% | 32 | 8% | -2.6% | -3.5% |
| North Center | 13% | 20% | 71 | 19% | 5.6% | -1.0% |
| Central | 17% | 18% | 49 | 13% | -4.2% | -4.9% |
| South East | 16% | 15% | 62 | 16% | 0.2% | 1.3% |
| East & North East | 19% | 20% | 67 | 18% | -1.5% | -2.6% |
| Sum | 100% | 100% | 382 | 100% | 0.0% | 0.0% |

**S9 Table.** Distribution of observations over the hours of the day

|  | Number PAs | Percentage |
| --- | --- | --- |
| Time | of PAs | of PAs |
| 10:00AM to 11:00AM | 23 | 6% |
| 11:00AM to 12:00PM | 50 | 13% |
| 12:00PM to 1:00PM | 62 | 16% |
| 1:00PM to 2:00PM | 54 | 14% |
| 2:00PM to 3:00PM | 57 | 15% |
| 3:00PM to 4:00PM | 59 | 15% |
| 4:00PM to 5:00PM | 44 | 12% |
| 5:00PM to 6:30PM | 33 | 9% |
| Total | 382 | 100% |

**S10 Table.** Distribution of observations over days of the week

|  |  | Number PAs | Percentage |
| --- | --- | --- | --- |
| Date | Day | of PAs | of PAs |
| 12/14/2020 | Monday | 44 | 12% |
| 12/15/2020 | Tuesday | 64 | 17% |
| 12/16/2020 | Wednesday | 51 | 13% |
| 12/17/2020 | Thursday | 68 | 18% |
| 12/18/2020 | Friday | 24 | 6% |
| 12/19/2020 | Saturday | 61 | 16% |
| 12/20/2020 | Sunday | 70 | 18% |
| Total | | 382 | 100% |

**S1 Fig.** Constructed city districts for Louisville, Kentucky


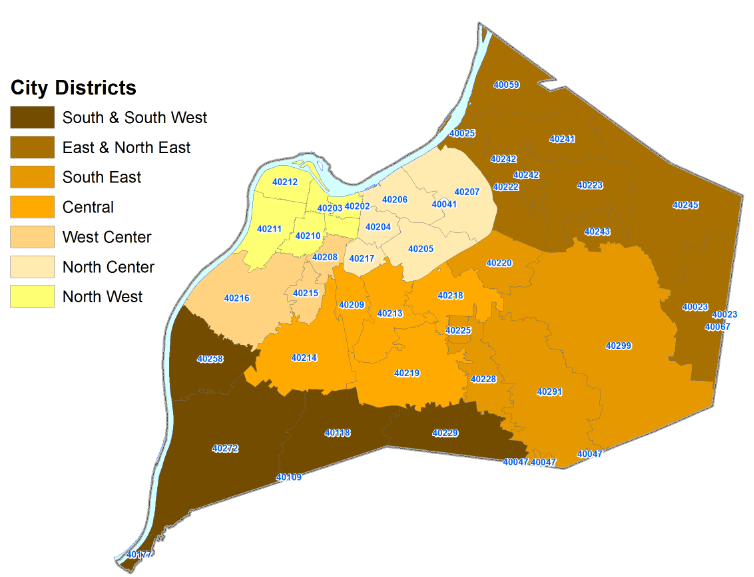


*Note:* This map is constructed by authors using ArcGIS Pro 2.7.1.

**S2 Fig.** Number of observed indoor public areas by city district

**71 (19%)**

**32 (8%)**

**67 (18%)**

**62 (16%)**

**49 (13%)**

**50 (13%)**

**51 (13%)**

**S3 Fig.** Number of observed indoor public areas by retail group

**71 (19%)**

**91 (24%)**

**133 (35%)**

**87 (23%)**

**S4 Fig.** Number of observed indoor public areas by size

**164 (43%)**

**82 (21%)**

**74 (19%)**

**62 (16%)**

**S5 Fig.** Percentage of perceived male-presenting persons among unmasked and incorrectly masked staff by district

| 1. % Male in Unmasked Staff |
| --- |
|  |
|  |
| 1. % Male in Incorrectly Masked Staff |
|  |

**S6 Fig.** Seven-day case and death rate per 100,000 in the United States and Louisville

| 1. Seven-day case rate per 100,000 |
| --- |
|  |
|  |
| 1. Seven-day death rate per 100,000 |
|  |
| *Note:* Author’s calculation using the New York Times COVID-19 case and death data (<https://github.com/nytimes/covid-19-data>). The seven-day case/death rate per 100,000 is the summation of cases/deaths in the last seven days per 100,000 of the population. |
