# Supplementary material for: Prevalence of unmasked and improperly masked behavior in indoor public areas during the COVID-19 pandemic: Analysis of a stratified random sample from Louisville, Kentucky
Source: PLoS One. 2021 Jul 28;16(7):e0248324. doi: 10.1371/journal.pone.0248324 (PMC8318281; doi:10.1371/journal.pone.0248324)
Supplement: Attachment — Submitted filename: Response to Reviewers.docx [file pone.0248324.s003.docx]

**Reviewer #1:**

The manuscript is well written and brings up an important topic in the current context. Yet, I have some comments for consideration by the authors.

1) Are there any results in terms of mask type that could be presented in the results section?

In a previous [survey](https://louisville.edu/sphis/documents-and-pdfs/ADAResultsofthesecondobservationalsurveysofmasksinindorpublicareasinjeffersoncountykyduringdec2020.pdf), albeit its structure and questions were different (most importantly, the questions were not designed to provide the mask use prevalence in percentage), we did include a question about the type of mask. At that time, we observed that 50% wore surgical masks and 50% wore cloth masks and 0-<1% wore face shields, bandanas, or neck gaiters. Also, we did not find any pattern (e.g., by district or PA size) in mask type. Therefore, we dropped that question from this survey as we needed to make subsequent surveys easier to conduct to prevent measurement error and more focused on measuring the mask non-use and incorrect use prevalences in percentage and identify a couple of demographics.

To address this comment, we have now added the following note to the last paragraph of the limitation section, lines 493-495 in the “Manuscript.docx”:

“The type of facial mask (e.g., surgical, cloth, bandana, gators, or face shields) was not observed in the current study. In the pilot survey,^55^ the predominant masks used were surgical (50%) and cloth masks (50%) that did not differ across public areas or zip codes.”

2) Why only up to ten visitors were observed in each PA? And which criteria did the observer use to choose the ten visitors that he/she observed in PAs with more than ten visitors? In other words, how did the observer avoid any bias in selecting the ten visitors he/she observed? It is important to clarify it, as an observer could observe different proportions of unmasked and masked visitors depending simply on the procedures adopted.

This is a very important question, and thank you for asking it. The reasons for observing a specific number of visitors or staff were (1) to establish a feasible and accurate denominator for the observance of unmasked or incorrectly masked prevalence rates and (2) to avoid selection bias.

Our experience from the previous [pilot survey](https://louisville.edu/sphis/documents-and-pdfs/ADAResultsofthesecondobservationalsurveysofmasksinindorpublicareasinjeffersoncountykyduringdec2020.pdf) ([link](https://louisville.edu/sphis/documents-and-pdfs/ObservationalSurveysofMaskUseandProtectiveMeasuresofIndoorPublicAreasWave1.pdf)) showed that counting all visitors and observing their mask use behavior in large public areas (e.g., a large department store such as Walmart) was not practical as it was challenging for surveyors to accurately recall all of the info on mask use, visitor/staff status, and perceived sex and age when filling the survey questionnaire. Therefore, we decided to observe a specific number of visitors and staff that was feasible for our surveyors and also more accurate.

We decided to observe up to 10 visitors and 10 staff for two major reasons:

1) We realized that it was easier for our typical surveyor to work with a multiple of 10 (e.g., 10, 20, 30, …) as the maximum number of visitors or staff observed than 11 or 21. Therefore, we thought of working with 10 or 20.
2) Before finalizing the questionnaire and training surveyors, we practiced taking surveys where 10 and 20 successive visitors or staff were observed (total 20 and 40 visitors and staff). Since the observance required memorizing numerous pieces of information (mentioned below in four steps), it was challenging to accurately keep track of all the information when more than 20 total people (total of visitors and staff) were observed.

*Note:* The process of observation included 4 steps:

(i) observing successive visitors (up to 10) and taking mental notes on their total count, the number of unmasked, the number of incorrectly masked, and perceived sex and age of the unmasked and incorrectly masked;
(ii) filling the visitors’ part of the questionnaire;
(iii) observing successive staff (up to 10) and taking mental notes on their total count, the number of unmasked, the number of incorrectly masked, and perceived sex and age of the unmasked and incorrectly masked;
(iv) filling the staff’s part of the questionnaire.

To address this comment, we have added a new paragraph to the text, under the subsection “The questionnaire and data collection method,” lines 265-276:

“The reason for observing a specific number of visitors or staff was to establish a denominator for the calculation of unmasked and incorrectly masked prevalence rates. The authors’ experience from a previous pilot observational survey of mask use showed that counting all visitors and observing their mask use behavior in large public areas were not practical.[^32^](https://ir.library.louisville.edu/jri/vol5/iss1/7/) Ten was selected as the maximum number of observed visitors or staff in a PA for two reasons. First, the practice surveys, conducted by three authors, showed that working with a multiple of 10 (e.g., 10 or 20) was more straightforward for a surveyor and less prone to error. Second, to select a multiple of 10, practice surveys that targeted the observation of 10 and 20 visitors or staff (maximum totals of 20 and 40 visitors and staff, respectively) were conducted. The results showed that keeping track of 40 (versus 20) people and memorizing several pieces of information (total count, the number of unmasked, the number of incorrectly masked, and perceived sex and age of the unmasked and incorrectly masked) was challenging.”

To avoid selection bias in the observation of visitors and staff, our surveyors started the observation of a PA as soon as they entered a PA. If the observation was not possible right after entering a PA, we asked surveyors to randomly select a location in the PA and start observing the first 10 people in the view from there. In other words, as soon as they started the observation, they were required to observe the first 10 people (visitors, then staff) consecutively. To address this comment, the second paragraph of the subsection “The questionnaire and data collection method”was rewritten to: (lines 253-264):

“In the second section, surveyors were asked to observe the mask use of up to ten visitors in the PA. If there were more than ten visitors, they needed to observe only ten of them, but if there were fewer than ten visitors, they needed to observe all of them. To avoid selection bias in the observation of visitors, the surveyors started the observation as soon as they entered the PA. If the observation was not possible right after entering the PA, they started the observation at a random time and location in the PA. In any case, they (1) did not start observation after seeing an unmasked or incorrectly masked visitor and (2) observed the first ten visitors in view. The second and third questions asked the numbers of the unmasked and incorrectly masked among the observed visitors, respectively. The fourth and fifth questions asked the perceived sex and age-ranges (0−18, 19−24, 25−44, 45−64, and 65+ years) of the unmasked and incorrectly masked. In the third section of the questionnaire, surveyors were asked about mask use among the PA staff. The section was structured similarly to the second section (Table 1).”

3) Was the use of facial mask mandatory throughout the period of observation? I suggest providing this information at some point of the introduction and discussion.

This is important information. Thank you for mentioning it. The use of facial masks was mandatory in all public spaces during the study period. The Governor of Kentucky issued an executive order that mandated wearing a facial covering in public places on July 9, 2020 ([link](https://governor.ky.gov/attachments/20200709_Executive-Order_State-of-Emergency.pdf)). This executive order has been reviewed was renewed until now ([link](https://governor.ky.gov/attachments/20210329_Executive-Order_2021-212_face-coverings-renewal.pdf)). Now we have added a note to the first paragraph of the introduction (lines 70-75) that points to the mandate:

“Transmission of respiratory viral infections like COVID-19 can be reduced considerably by using a facial mask, especially in indoor public areas.^1-8^ Therefore, as COVID-19 became a widespread pandemic in 2020, countries, states, and local municipalities began requiring facial coverings. Many governors within the United States implemented executive orders requiring masks within public indoor areas throughout 2020.^9^ The state of Kentucky mandated public masking on July 9th, 2020, and in March 2021 renewed the executive order until at least July 2021.^10,11”^

4) What was the proportion of PAs that presented reinforcement signs? And did the existence of these reinforcement signs presented any influence on the use of facial mask?

In the previous [survey](https://louisville.edu/sphis/documents-and-pdfs/ADAResultsofthesecondobservationalsurveysofmasksinindorpublicareasinjeffersoncountykyduringdec2020.pdf) (Figures 4-10), we extensively assessed COVID-19 protection measures at the observed PAs. We included two questions about the availability of hand sanitizer for visitors and mask reinforcement signs at the PA. We did not observe a significant difference in the share of PAs with mask use reinforcement signs across the city (See Figure 12 in the technical report that preceded this manuscript, [LINK](http://louisville.edu/sphis/documents-and-pdfs/ADAResultsofthesecondobservationalsurveysofmasksinindorpublicareasinjeffersoncountykyduringdec2020.pdf)), although mask use behavior was different in different districts that prompted the current study. We did not report these pilot results in the current manuscript for two reasons: (1) We did not plan on including these questions in the next waves of the survey, and (2) We intended to make the focus of this study on developing a method to measure the prevalence of mask use and its correct use.

**Reviewer #2:**

**## Summary**

The manuscript showed data about the use (prevalence of incorrect use) of masks during the COVID-19 pandemic period at the end of 2020. Previous studies have been suggested low levels of incorrectly used masks because of self-reported procedures. This study presented data that may represent better the “real world” scenario since it used an observational evaluation and not a self-reported procedure. I recommend accepting this manuscript after minor revision. Some comments and suggestions are described below:

**## Comments on abstract and title**

**#1.** Although the method is a great part of the manuscript, the information about the incorrect use of masks may be the most relevant news from the study. In this way, I suggest authors change the title for “Prevalence of Unmasked and Improperly Masked in Indoor Public Areas during COVID-19 Pandemic: Data From Stratified Random Sampling Methodology For Observing Community”.

Thank you very much. We agree with you that the current title makes the paper sound like it is about sampling methodology, not about mask behavior. Therefore, in line with your suggestion, we changed to:

“Prevalence of Unmasked and Improperly Masked Behavior in Indoor Public Areas during the COVID-19 Pandemic: Analysis of a Stratified Random Sample from Louisville, Kentucky”

Lines 1-3 in the “Manuscript.docx”.

**#2.** The abstract is clear and presented the lack of information required in the field specifically solved in this manuscript (prevalence of incorrect use of masks is different from observational studies than self-declaration studies). In the abstract, it was informed that “The average mask use prevalence among observed visitors of the 382 visited public areas was 96%, while the average prevalence of proper use was 86%.”. It may be relevant to inform the total number of subjects observed in these 382 public areas.

Of course! We observed 3,590 persons in this study: 2,080 visitors and 1510 staff. We have reported these numbers in the abstract (lines 57-58) and the results section (lines 288-289).

**## Comments on introduction/background**

**#3.** The authors presented the background and the relevance of the study in the introduction adequately. The authors presented the arguments about the use of an observational procedure instead of a questionary to collected data from subjects about the correct use of masks. However, I recommend showing at least some results about the prevalence of incorrect use of masks from the studies cited in the introduction.

We have found only one study that measured the incorrect use of a mask. That was a PLOS ONE study by Haischer et al. (2020):

Haischer MH, Beilfuss R, Hart MR, et al. Who is wearing a mask? Gender-, age-, and location-related differences during the COVID-19 pandemic. PLOS ONE. 2020;15(10):e0240785.

We have mentioned this study in the introduction and reported its results (Lines 83-86):

“Observational studies that do not focus on a specific sub-population or improper masking are scarce.^22^ A study conducted in Wisconsin, USA, in summer 2020 approximated a 6% improper mask-wearing rate before the state’s store mask mandate and suggested it decreased to 3% with a store mandate.^23^”

**## Comments on methods**

**#4.** For better replicability of this study in other cities and countries, I recommend the author inform the number and profile of the volunteers/observators. Were they students from university? Were they wearing special protection? Were they vaccinated before starting the field procedure? Some of these questions also imply the ethical aspects of the involvement of the volunteers.

Thank you for this great point. We added a subsection (Surveyors’ backgrounds and training) to the materials and methods section and described the surveyors. The description includes details about the year of study for student surveyors, compensation, and protective measures (Lines 216-225).

This new subsection includes some of the information from a previous subsection (namely, The Survey Implementation) as well.

“This study employed ten trained surveyors. Five of the surveyors were undergraduate students, 4 were public health doctoral students, and 1 faculty member from the university conducting the study. Among the group, 4 were men and 6 were women. All participated in a 1 hour orientation training prior to observation to ensure uniform collection efforts. Training included a review of the definitions of a mask, an unmasked person, and an incorrectly masked person and instructions for personal protective equipment use, conducting observations, and instructions on data entry. All received surgical masks, hand sanitizer, and face shields to protect them during observation. None of the surveyors were vaccinated prior to observation. Surveyors received monetary compensation for hours observing and mileage accrued during observations.”

**#5.** What was the period of the day (morning, afternoon…) of the observation. The profile of visitors may be different in the early morning in comparison with the end of the day.

The study was conducted from 10:00 AM to 6:00 PM. Surveyors were asked to distribute their observations evenly among different hours of the day and different days of the week. The distribution of observations over days of week and hours, presented in the below tables, seem fairly even.

Except for the earliest and the latest observation hours (10:00 AM to 11:00 AM and 5:00 PM to 6:30 PM, respectively) in which 6% and 9% of the observations were made, the share of other hours in total observations was between 12% and 16%. 3 PAs were observed between 6:00 PM and 6:30 PM. A completely even distribution would have resulted in observing 12.5% of the 382 PAs per hour (below table).

Distribution of observations over times of the day

| Time | # of PAs | % of PAs |
| --- | --- | --- |
| 10:00AM to 11:00AM | 23 | 6% |
| 11:00AM to 12:00PM | 50 | 13% |
| 12:00PM to 1:00PM | 62 | 16% |
| 1:00PM to 2:00PM | 54 | 14% |
| 2:00PM to 3:00PM | 57 | 15% |
| 3:00PM to 4:00PM | 59 | 15% |
| 4:00PM to 5:00PM | 44 | 12% |
| 5:00PM to 6:30PM | 33 | 9% |

Also, except for Friday, in which 6% of the 382 PAs were observed, the share of other days of the week in the total observed PAs were between 12% and 18%. Also, 34% of the PAs were observed on a weekend day, 66% on a weekday. A completely even distribution would have resulted in 29% weekend, 71% weekday (below table).

Distribution of observations over days of the week

| Date | Day | # of PAs | % of PAs |
| --- | --- | --- | --- |
| 12/14/2020 | Monday | 44 | 12% |
| 12/15/2020 | Tuesday | 64 | 17% |
| 12/16/2020 | Wednesday | 51 | 13% |
| 12/17/2020 | Thursday | 68 | 18% |
| 12/18/2020 | Friday | 24 | 6% |
| 12/19/2020 | Saturday | 61 | 16% |
| 12/20/2020 | Sunday | 70 | 18% |

These results are added to the supplementary materials in Supplementary Tables 8 and 9. Also, we added the following note to the end of the results subsection titled “Sample characteristics” (lines 309-315):

“The distributions of observations were fairly even throughout days of the survey week and hours of the days (S8,S9 Tables). Except for Friday (December 18, 2020) in which 6% of the 382 PAs were observed, the share of other days of the week in the total observed PAs were between 12% and 18%. About 34% of the PAs were observed on a weekend day, 66% on a weekday. Except for the earliest and the latest observation hours (10:00 AM to 11:00 AM and 5:00 PM to 6:30 PM, respectively) in which 6% and 9% of the observations were made, the share of other hours in total observations was between 12% and 16%.”

**#6.** The authors informed that “The fourth and fifth questions asked the surveyor’s to perceive the sex and approximate age-ranges”. There was a previous training procedure to prepare the surveyors to identify the age of the subjects? Is there some limitations fro this identification because of the use of masks?

Thank you for the question. This is a limitation of this study and can result in an observer bias as the surveyors could have misjudged the sex and the approximate age ranges of the staff and visitors because of the use of masks. There was no formal training provided to the surveyors in determining the age of the staff or visitors. We have added a related note to the limitation section of the manuscript, lines 488-492:

“… In other words, the study introduced observer bias as a product of misjudging visitors’ and staff’s sex and age due to limited facial visibility. As a result, sex and age could have been misclassified to some degree. In addition, misclassification of mask use or correct use could have occurred if an observed visitor or staff member took off or moved face-covering temporarily during observation.”

**## Comments on results**

**#7.** The results are clear and well presented. I suggest to authors to first present the “Mask wearing for public areas’ visitors” results (page 14) following by the “Mask wearing for public areas’ staff” (page 15) and after that to present the results described now on page 13.

Thank you for your comment. As you mentioned, the core results are presented in subsections “Mask wearing for public areas’ visitors” and “Mask wearing for public areas’ staff” of the results section. What is presented before these two subsections is a description of the sample. We have presented these materials first to provide an assessment of the reliability of the data collection. Given this background, we move on to providing the main results. In the revised manuscript, we have given a title to these materials: “Sample characteristics,” line 287.

**## Comments on discussion**

**#8.** Since the adhesion to health recommendation is influenced by many aspects, from political recommendations procedures, level of understanding of the population, media coverage, self-perception of risk, economic and educational status, I suggest to authors include some aspects in the discussion. It is clear the impact of the position of some governments, mainly in Brazil and USA, on the use of masks. I suggest including this scenario in the manuscript. Also, I suggest reading two references:

Taylor S. The psychology of pandemics: Preparing for the next global outbreak of infectious disease. Newcastle upon Tyne: Cambridge Scholars Publishing. Cambridge Sch. 2019.

This reference discusses many aspects that may interfere in the adhesion of health recommendations. Some parts of this discussion are also present in the discussion of this paper:

Heck et al., Insufficient social distancing may contribute to COVID-19 outbreak: The case of Ijuí city in Brazil. PLoS One. 2021 Feb 17;16(2):e0246520. <https://doi.org/10.1371/journal.pone.0246520>

Thank you for this feedback. We agree with your point that the factors influencing behavior are complex and multidimensional. We reviewed the two references you provided. To expand our discussion to address the psychological factors, we added a paragraph to discuss this. We located this discussion paragraph after the discussion on risk (lines 436-443):

“The psychological factors that influence adherence to recommendations for public health behaviors often receive less attention than the medical factors. Although medical and public health practices have evolved throughout the pandemics of the past, human psychology has adapted less.^48^ Attending to the psychological factors, such as those that lead individuals to engage in risky behaviors or send others into extreme isolation, warrant public health planning as well.^48,49^ Individuals’ anxiety and beliefs about their own health influence adherence to hygiene practices and social distancing.^48^ Future pandemic planning will need to consider planning for increased psychological needs and crises.”

To expand the discussion about the geopolitical factors that influence adherence, we added to the last paragraph of the discussion (lines 444-447 and lines 454-457) to improve the manuscript:

Lines 444-447: “Political factors, cultural dynamics within a community, socioeconomic factors, media and social media, and governmental policies influence a community’s adherence to health behavior recommendations.”

Lines 454-457: “Further, the evidence from international responses’ also highlights how political dynamics and governmental policies impact a population’s response to pandemic health recommendations.^53^ Even more localized leadership, beyond a national or state level, can influence a community’s response.^53^”

**Reviewer #3:**

**Summary:** The study under review explored a method for measuring the prevalence of mask-wearing and proper mask use in indoor public areas without relying on self-report. A stratified random sample of retail trade stores (public areas) in Louisville was selected and targeted for observation by trained surveyors during December 14−20, 2020. The stratification allowed for investigating mask use behaviour by city district, retail trade group, and public area size. The authors noted a high average mask use prevalence (96%) among observed visitors of the 382 visited public areas, with an average prevalence of proper use of 86%. The authors also noted a high average mask use prevalence (92%) among staff, with unmasked staff being observed in fewer public areas. Observing unmasked and incorrectly masked visitors were more common in smaller public places and food and grocery stores. The majority of the observed unmasked persons were male and middle age adults. The authors suggested a continued need to improve awareness of the effectiveness of appropriate facial mask use, financial resources to provide masks, particularly in low-income areas, and education on correct ways to wear a mask.

The authors are commended for a well-written manuscript. The arguments for the manuscript under review are timely and original. I believe there are minor concerns and issues with the manuscript in its current form that need to be addressed before being considered for publication. All my comments are included below. I hope you will find them to be constructive and helpful. The primary concerns with the manuscript are presented below:

**Introduction.** The Introduction section is well-written but relatively short in general. The authors are encouraged to bring out the main points a little more firmly and insert necessary information. The authors are advised, for example, to include a priori research hypotheses with their respective references.

Thank you for this comment. We have expanded the article’s introduction now. Specifically, we have provided information on

- the timing of mask mandates in the united states and the state of Kentucky,
- further discussion on the effectiveness of mask-wearing in curbing the spread of the coronavirus,
- prior research that measured mask use behavior (especially one study that measured the proper use of masks), and
- the timing of this study and what it measures.

Lines 69-97 in the “Manuscript.docx”. It reads as following now:

“Transmission of respiratory viral infections like COVID-19 can be reduced considerably by using a facial mask, especially in indoor public areas.^1-8^ Therefore, as COVID-19 became a widespread pandemic in 2020, countries, states, and local municipalities began requiring facial coverings. Many governors within the United States implemented executive orders requiring masks within public indoor areas throughout 2020.^9^ The state of Kentucky mandated public masking on July 9th, 2020, and in March 2021 renewed the executive order until at least July 2021.^10,11^

The requirement for facial coverings potentially limits the spread of the virus by asymptomatic and pre-symptomatic individuals who may cause nearly 60% of COVID-19 cases.^12,13^ While the importance of mask-wearing is emphasized, understanding mask-wearing behavior and practices in the community can inform public health policies. However, common methods of measuring mask-wearing yield significant inaccuracies, particularly surveys based on self-report^14-18^ as they result in attenuation bias. When non-interventional observations of mask use replace self-reporting, non-representative sub-populations (e.g., university students and clinic population) have been studied.^19-21^ Observational studies that do not focus on a specific sub-population or improper masking are scarce.^22^ A study conducted in Wisconsin, USA, in summer 2020 approximated a 6% improper mask-wearing rate before the state’s store mask mandate and suggested it decreased to 3% with a store mandate.^23^

This study’s primary aim was to develop a method to measure mask-wearing behavior in public areas accurately. To this purpose, visitors and staff of a representative sample of indoor public areas (PAs) in the city of Louisville (estimated 2019 population: 766,757),^24^ Kentucky, USA, were observed. The representativeness of PAs was ensured by a stratified random sampling method, which allowed for assessing disparities in mask usage across city districts, industries, and PA sizes.

Also, in contrast to most existing mask usage studies,^14-22^ this study measured the prevalence of proper facial mask use in addition to the prevalence of facial mask use, as the effectiveness of mask mandates will be limited if a mask is not used properly. This study does not measure the prevalence of mask use and proper use before and after Kentucky’s statewide mask mandate on July 9, 2020. Instead, it sought to identify the prevalence rate in December 2020 at the peak of the pandemic in the United States (S1 Fig).”

**Materials and Methods.** The study was conducted for a brief period of 2020 December. Could the study findings, therefore, be similar to other periods? Please comment. Additionally, how was the COVID-19-related scenario (n. of cases and n. of deaths) in the USA and Louisville area during the study? The study’s results may, at least in part, depend on the pandemic scenario in the city/country. The authors are, therefore, suggested to take into account this inherent “limitation.” The authors are also advised to insert a figure depicting the COVID-19 milestones, which could help the readers. Is there any explanation/reason to observe 10 visitors in each PA? Please comment.

These are important considerations, and thank you for bringing them up. In the following, we have explained how we have addressed them in two parts.

**COVID-19 trends in the Louisville area and its implications for the findings of this study**

If personal protection behavior is affected by the extent of the pandemic in the country and their community, one would expect mask use behavior to be different at times with varying rates of infections and deaths from COVID-19. In fact, this survey was conducted at the height of the COVID-19 pandemic in Louisville and the United States (See the newly added figure, Supplementary Figure 1). Therefore, we might be measuring the highest mask use and proper use rates in Louisville. Acknowledging this important point (i.e., the dynamics of mask use behavior), we have added a note to the introduction and limitation sections of the paper (lines 94-97 and lines 478-483):

Lines 94-97: “This study does not measure the prevalence of mask use and proper use before and after Kentucky’s statewide mask mandate on July 9, 2020. Instead, it sought to identify the prevalence rate in December 2020 at the peak of the pandemic in the United States (S1 Fig).”

Lines 478-483: “The survey of mask use behavior was conducted at the height of the COVID-19 pandemic in the United States and the city of Louisville (S1 Fig). If people’s personal protection behavior is affected by the extent of the pandemic in the country and their community, one would expect that mask use behavior to be different at times with varying rates of infection and deaths from COVID-19. Therefore, the results of this study may not capture mask use behavior of citizens in other periods.”

**Why observing 10 visitors in each PA**

This is a very important question, and thank you for asking it. The reasons for observing a specific number of visitors or staff were (1) to establish a feasible and accurate denominator for the observance of unmasked or incorrectly masked prevalence rates and (2) to avoid selection bias.

Our experience from the previous [pilot survey](https://louisville.edu/sphis/documents-and-pdfs/ADAResultsofthesecondobservationalsurveysofmasksinindorpublicareasinjeffersoncountykyduringdec2020.pdf) ([link](https://louisville.edu/sphis/documents-and-pdfs/ObservationalSurveysofMaskUseandProtectiveMeasuresofIndoorPublicAreasWave1.pdf)) showed that counting all visitors and observing their mask use behavior in large public areas (e.g., a large department store such as Walmart) was not practical as it was challenging for surveyors to accurately recall all of the info on mask use, visitor/staff status, and perceived sex and age when filling the survey questionnaire. Therefore, we decided to observe a specific number of visitors and staff that was feasible for our surveyors and also more accurate.

We decided to observe up to 10 visitors and 10 staff for two major reasons:

1) We realized that it was easier for our typical surveyor to work with a multiple of 10 (e.g., 10, 20, 30, …) as the maximum number of visitors or staff observed than 11 or 21. Therefore, we thought of working with 10 or 20.
2) Before finalizing the questionnaire and training surveyors, we practiced taking surveys where 10 and 20 successive visitors or staff were observed (total 20 and 40 visitors and staff). Since the observance required memorizing numerous pieces of information (mentioned below in four steps), it was challenging to accurately keep track of all the information when more than 20 total people (total of visitors and staff) were observed.

*Note:* The process of observation included 4 steps:

(i) observing successive visitors (up to 10) and taking mental notes on their total count, the number of unmasked, the number of incorrectly masked, and perceived sex and age of the unmasked and incorrectly masked;
(ii) filling the visitors’ part of the questionnaire;
(iii) observing successive staff (up to 10) and taking mental notes on their total count, the number of unmasked, the number of incorrectly masked, and perceived sex and age of the unmasked and incorrectly masked;
(iv) filling the staff’s part of the questionnaire.

To address this comment, we have added a new paragraph to the text, under the subsection “The questionnaire and data collection method,” lines 265-276:

“The reason for observing a specific number of visitors or staff was to establish a denominator for the calculation of unmasked and incorrectly masked prevalence rates. The authors’ experience from a previous pilot observational survey of mask use showed that counting all visitors and observing their mask use behavior in large public areas were not practical.[^32^](https://ir.library.louisville.edu/jri/vol5/iss1/7/) Ten was selected as the maximum number of observed visitors or staff in a PA for two reasons. First, the practice surveys, conducted by three authors, showed that working with a multiple of 10 (e.g., 10 or 20) was more straightforward for a surveyor and less prone to error. Second, to select a multiple of 10, practice surveys that targeted the observation of 10 and 20 visitors or staff (maximum totals of 20 and 40 visitors and staff, respectively) were conducted. The results showed that keeping track of 40 (versus 20) people and memorizing several pieces of information (total count, the number of unmasked, the number of incorrectly masked, and perceived sex and age of the unmasked and incorrectly masked) was challenging.”

**Discussion.** Face-covering misclassification could be an inherent limitation of this observational study. As mentioned by the authors, both sex and age could have been misclassified due to surveyor bias. Face-covering misclassification, however, could also occur if the mask was removed immediately before the observation. This potential scenario should, at a minimum, be considered by the authors.

Misclassification of sex and age is possible in this study. Now, we have acknowledged this issue by adding a note at the end of the limitation section (lines 488-483):

“… In other words, the study introduced observer bias as a product of misjudging visitors’ and staff’s sex and age due to limited facial visibility. As a result, sex and age could have been misclassified to some degree. In addition, misclassification of mask use or correct use could have occurred if an observed visitor or staff member took off or moved face-covering temporarily during observation.”
